# Primary extranodal natural killer/T-cell nasal-type lymphoma of spine: A case report

**DOI:** 10.1097/MD.0000000000017661

**Published:** 2019-11-01

**Authors:** Jie Wang, Ning Li, Yonggang Fan, Ningning Yang, Lei Xia

**Affiliations:** Department of Orthopedics, The First Affiliated Hospital of Zhengzhou University, Zhengzhou, China.

**Keywords:** Epstein–Barr virus, nasal type, NK/T-cell lymphoma, thoracic vertebra

## Abstract

**Rationale:**

Primary Extranodal Natural Killer/T-Cell Nasal-Type Lymphoma (ENKTCL) of spine is rarely reported. This case study presents a rare case of ENKTCL originating from the sixth thoracic vertebra.

**Patient concerns:**

Here, we present a case of 49-year-old Asian male with chest and back pain. Physical examination revealed the myodynamia of both lower limbs decreased from IV degree to 0 degree, reflexes of both lower limbs and a large area of cutaneous sensation below xiphoid process disappeared in 3 days.

**Diagnoses:**

In immunophenotype analysis, CD2, CD3, CD7, and CD68 were positive and CD56 was suspiciously positive. Granzyme B and T-cell intracellular antigen (TIA-1) were also positive and in situ hybridization was positive for Epstein–Barr virus–encoded mRNA (EBER). Ki-67 was 60%+. Nuclide bone scan showed that the nuclide was unusually concentrated in the sixth thoracic vertebra which considered extremely active and slightly concentrated in the right sacrolilac joint. Magnetic resonance imaging detected an abnormal signal in the sixth thoracic vertebra with corresponding paravertebral and intraspinal occupying lesion. Based on the above features, a diagnosis of ENKTCL was made.

**Interventions:**

This patient was treated with surgery and symptomatic supportive treatment.

**Outcomes:**

The myodynamia of patient's both lower limbs were elevated to I degree after the operation with chest and back pain partly relieved. However, the patient died about 3 months later.

**Lessons:**

ENKTCL could originate from spine. Clinicians should be alert for early stage diagnose and distinguish it from some common spinal tumor such as neurofibroma and hemangioma.

## 1 Introduction

Extranodal NK/T-cell lymphoma, nasal type (ENKTCL), previously known as lethal midline granuloma is a distinct clinic-pathological entity associated with Epstein–Barr virus that typically causes destruction of the midface, palatal, and orbital walls. In addition, ENKTCL can involve the skin, soft tissue, testes, gastrointestinal, and upper respiratory tract.^[1]^ To our limited knowledge, ENKTCL arising primarily from the spine without nasal involvement is distinctly rare with only 1 patient clearly documented in the literature.^[2]^

Diagnosis of such cases may be challenging due to the lower prevalence. However, because this kind of non-Hodgkin lymphoma is aggressive, early diagnosis and treatment is crucial. We herein report a very rare case of ENKTCL that have a primary origin from the sixth thoracic vertebra and describe the details of diagnosis and treatment.

## 2 Case report

**Figure 1:**
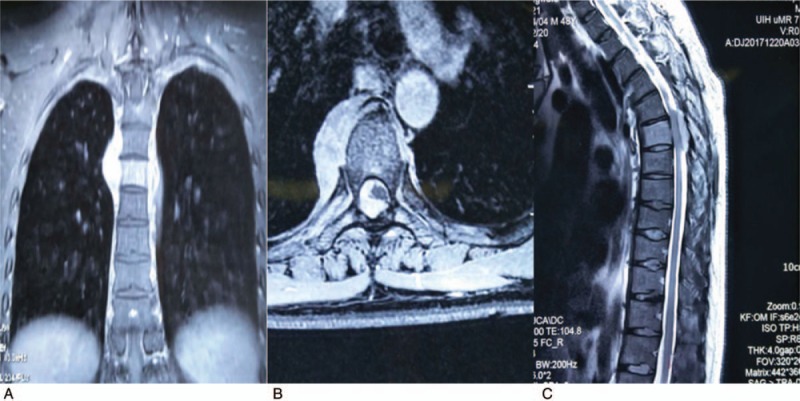
(A–C) MRI revealed an abnormal signal in the sixth thoracic vertebra with corresponding paravertebral and intraspinal occupying lesion that was considered as a suspected case of neurofibroma or hemangioma (A: coronal view, B: axial view, C: sagittal view).

A 49-year-old male, who suddenly suffered from chest and back pain, meanwhile, had slight paralysis below xiphoid process on June, 2017. Then, he took no special treatment except a magnetic resonance imaging in the Central Hospital of Zhoukou City, Henan Province, China on December 20, which revealed an abnormal signal in the sixth thoracic vertebra with corresponding paravertebral and intraspinal occupying lesion that was considered as a suspected case of neurofibroma or hemangioma (Fig. 1A–C).

**Figure 2:**
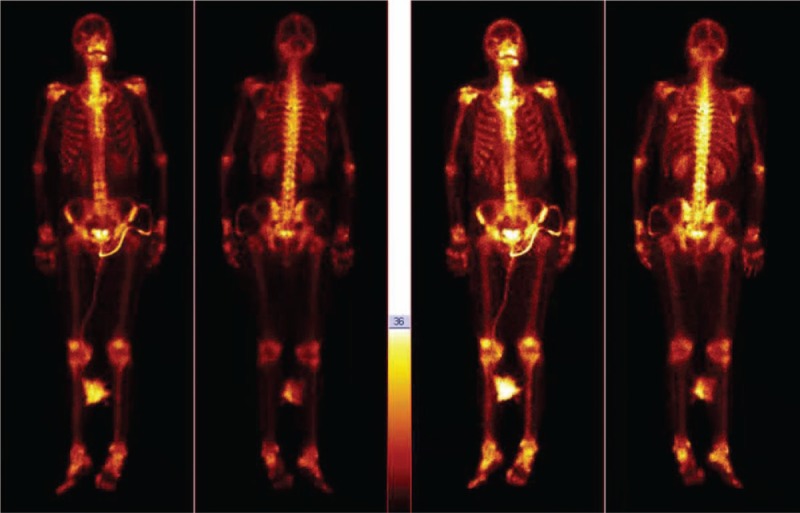
Nuclide bone scan showed that the nuclide abnormally concentrated in the sixth thoracic vertebra and slightly concentrated in the right sacrolilac joint.

**Figure 3:**
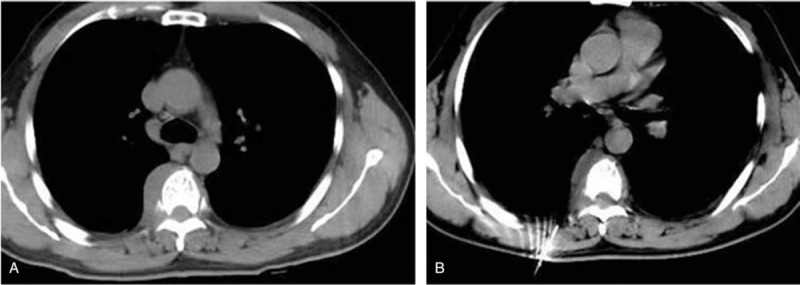
(A and B) Enhanced CT scan and bone marrow biopsy of paravertebral tissue guided by CT.

On December 29, 2017, the patient was admitted to our institute without any facial abnormalities such as swelling, purulent discharge or epistaxis from bilateral nostril. He denied any fever, night sweats, headache, dyspnea, dysphagia, and weight loss. Physical examination revealed the myodynamia of both lower limbs decreased from IV to 0 degree within 3 days, and the reflex of both lower limbs and a large area of cutaneous sensation below xiphoid process disappeared at the same time. Routine haematological and biochemical tests showed the percentage of lymphocytes and neutrophil were 16% and 13.7%, respectively. Tuberculosis specific secretion antigen was positive, however anti-mycobacterium tuberculosis IgM and IgG were negative. Nuclide bone scan showed that the nuclide abnormally concentrated in the sixth thoracic vertebra and slightly concentrated in the right sacrolilac joint (Fig. 2A and B). Enhanced computer tomography (CT) scan revealed obvious soft-tissue mass around the sixth thoracic vertebra and the bone marrow biopsy of paravertebral tissue guided by CT showed granulomatous inflammation accompanied by necrosis (Fig. 3A and B). Cytopathology of the paravertebral tissue showed a small amount of lymphocyte and neutrophil. Tubercle bacillus DNA detection and acid-fast bacillus immunohistochemistry staining were both negative.

It appeared as a tumor of the sixth thoracic vertebra but we were unable to confirm its nature. Considering the rapid increase of nerve compression symptoms, we consulted with doctors in the department of thoracic surgery and decided to take a posterior approach for excision of the sixth thoracic vertebral paravertebral as well as intraspinal occupying lesion. Finally, we used pedicle screw internal fixation system and bone cement to reconstruct the stability of spine and conducted a lesion pathological evaluation.

In immunophenotype analysis, CD2, CD3, CD7, and CD68 were positive and CD56 was suspiciously positive (Fig. 4  ). However, other T-cell antigens such as CD1a, CD4, CD5, CD8, and B-cell antigens such as CD20 and CD79a were negative. In addition, granzyme B and TIA-1 (T-cell intracellular antigen) were also positive and in situ hybridization was positive for Epstein–Barr virus–encoded mRNA (EBER). Meanwhile, Ki-67 was 60%+, indicating a high cell proliferation (Fig. 5  ). *TCR* gene rearrangement revealed TCRB, TCRG, and TCRD were negative, confirming no monoclonal proliferative T lymphocyte group. Based on the above features, a diagnosis of ENKTCL was made.

**Figure 4:**
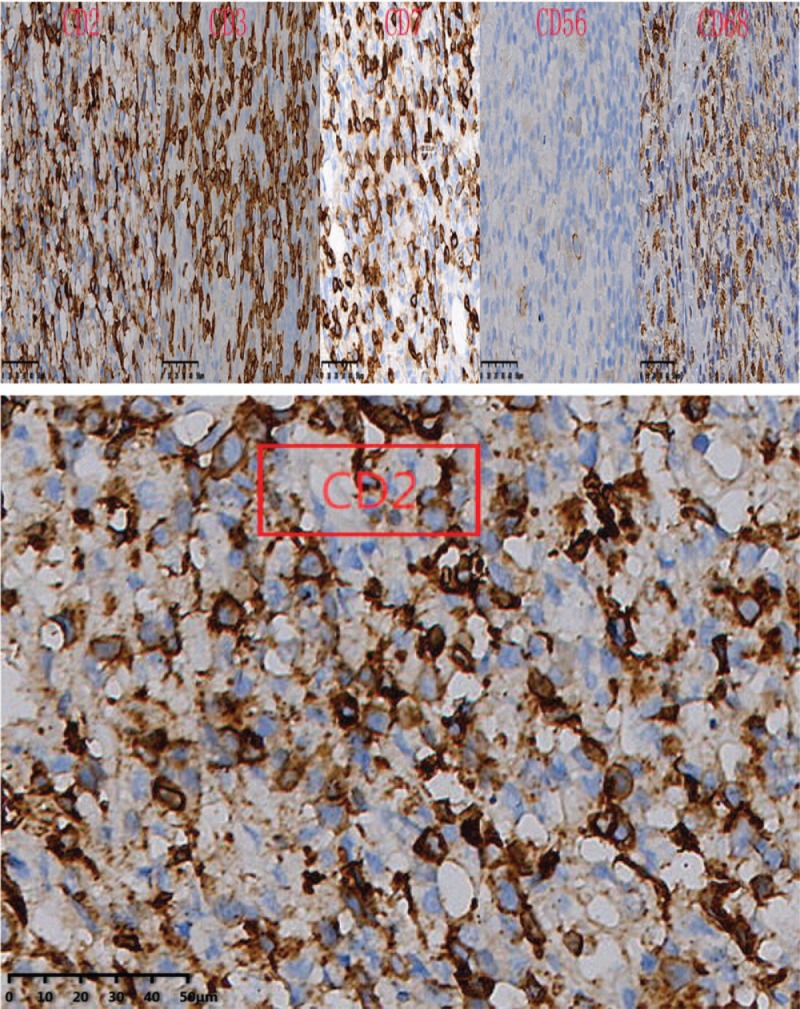
In immunophenotype analysis, CD2, CD3, CD7, and CD68 were positive and CD56 was suspiciously positive (40×).

**Figure 4 (Continued):**
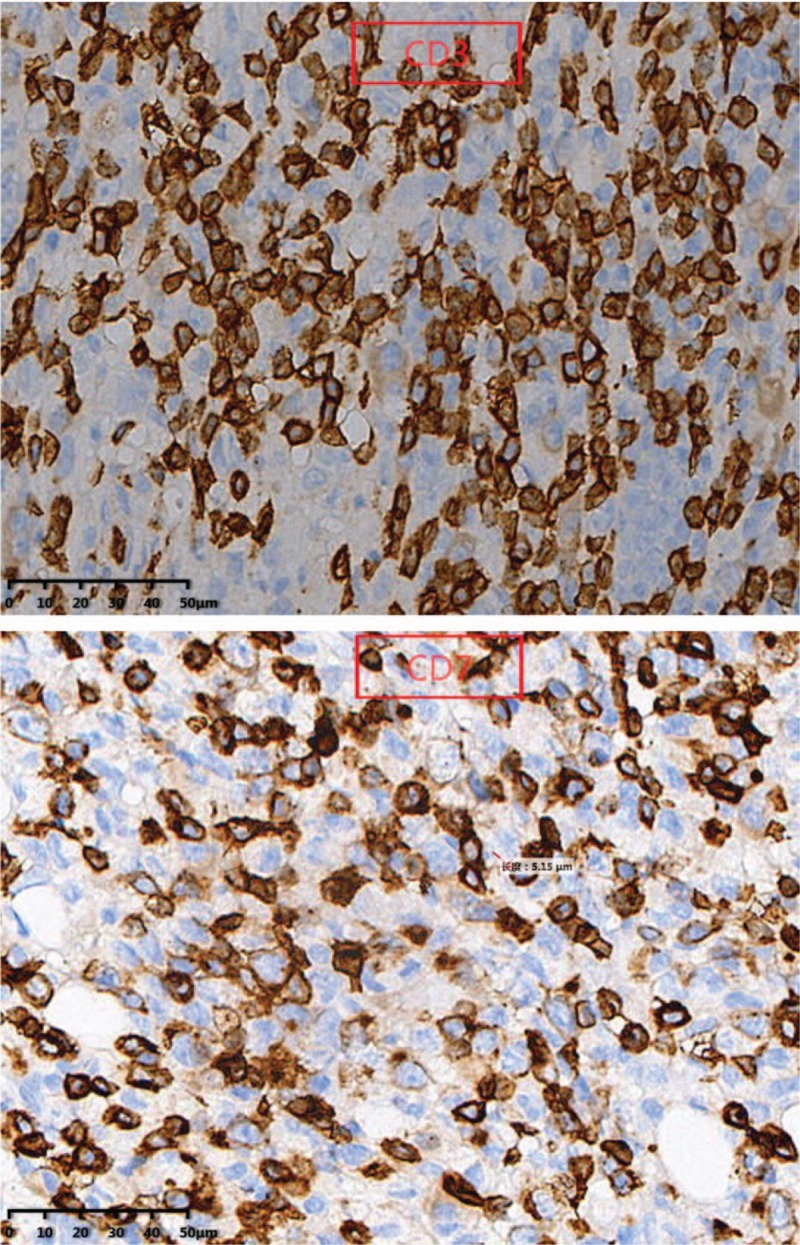
In immunophenotype analysis, CD2, CD3, CD7, and CD68 were positive and CD56 was suspiciously positive (40×).

**Figure 4 (Continued):**
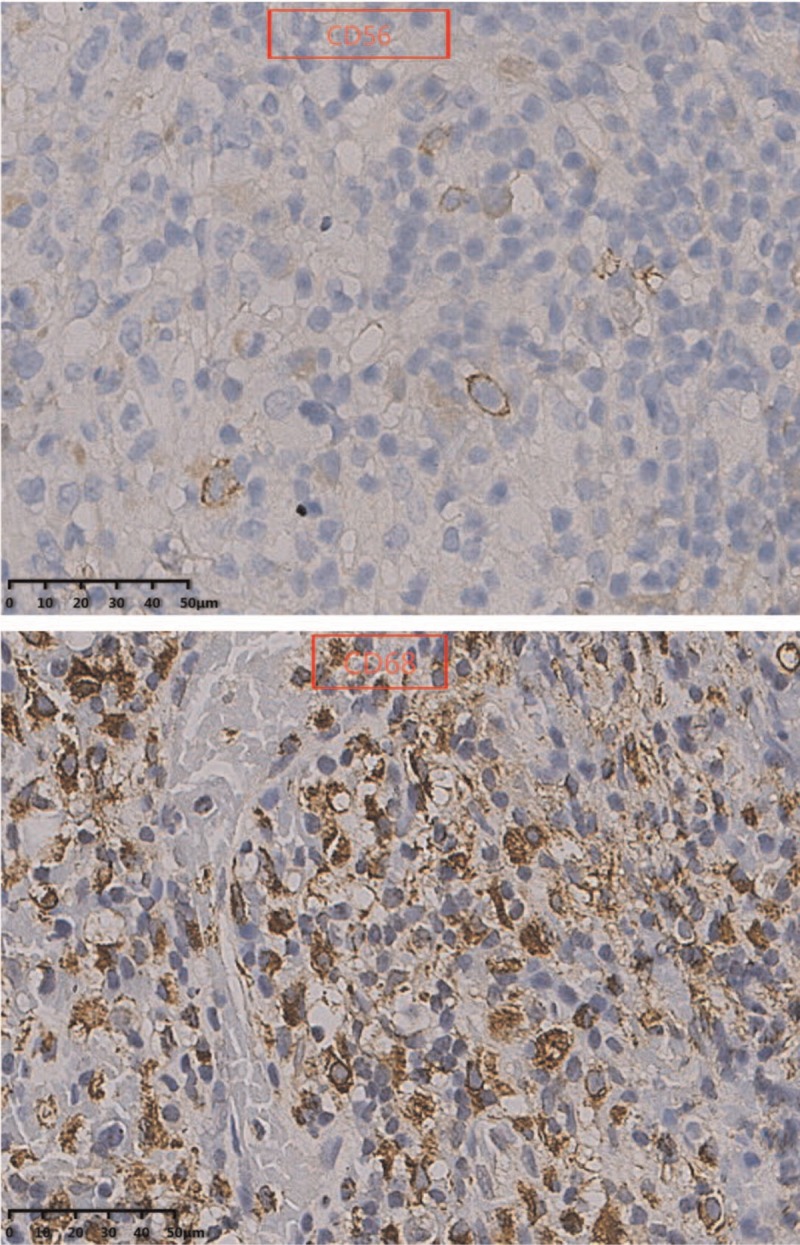
In immunophenotype analysis, CD2, CD3, CD7, and CD68 were positive and CD56 was suspiciously positive (40×).

**Figure 5:**
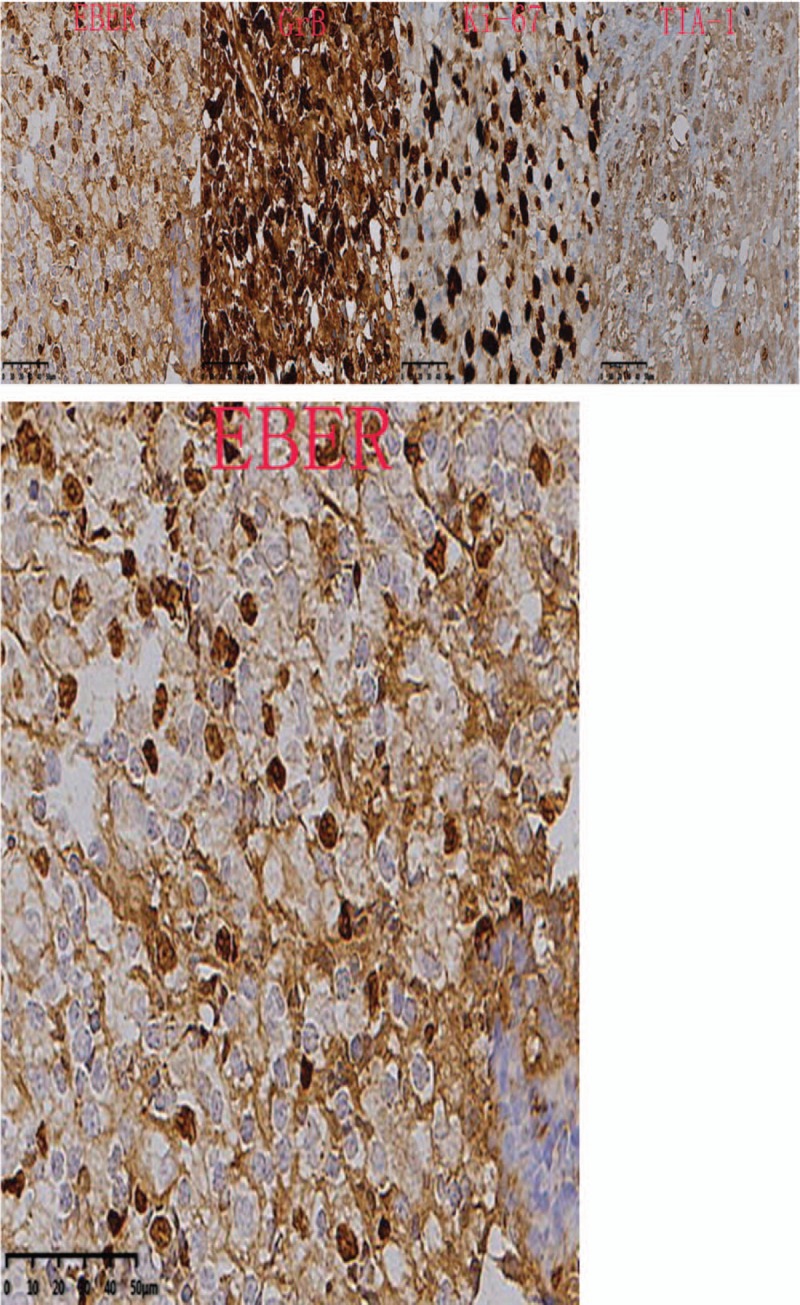
Granzyme B, TIA-1 (T-cell intracellular antigen) and Epstein–Barr virus–encoded mRNA (EBER) were positive. Ki-67 was 60%+, which indicated high cell proliferation (40×).

**Figure 5 (Continued):**
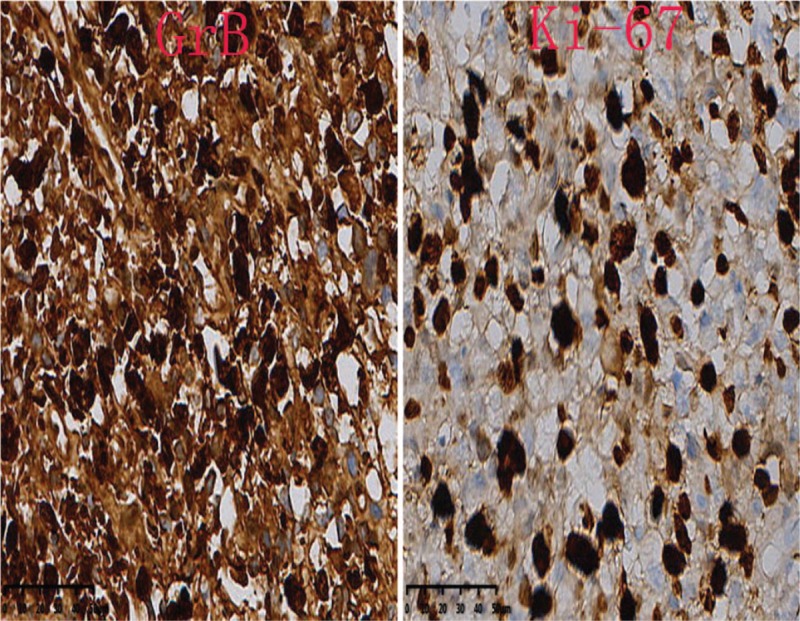
Granzyme B, TIA-1 (T-cell intracellular antigen) and Epstein–Barr virus–encoded mRNA (EBER) were positive. Ki-67 was 60%+, which indicated high cell proliferation (40×).

**Figure 5 (Continued):**
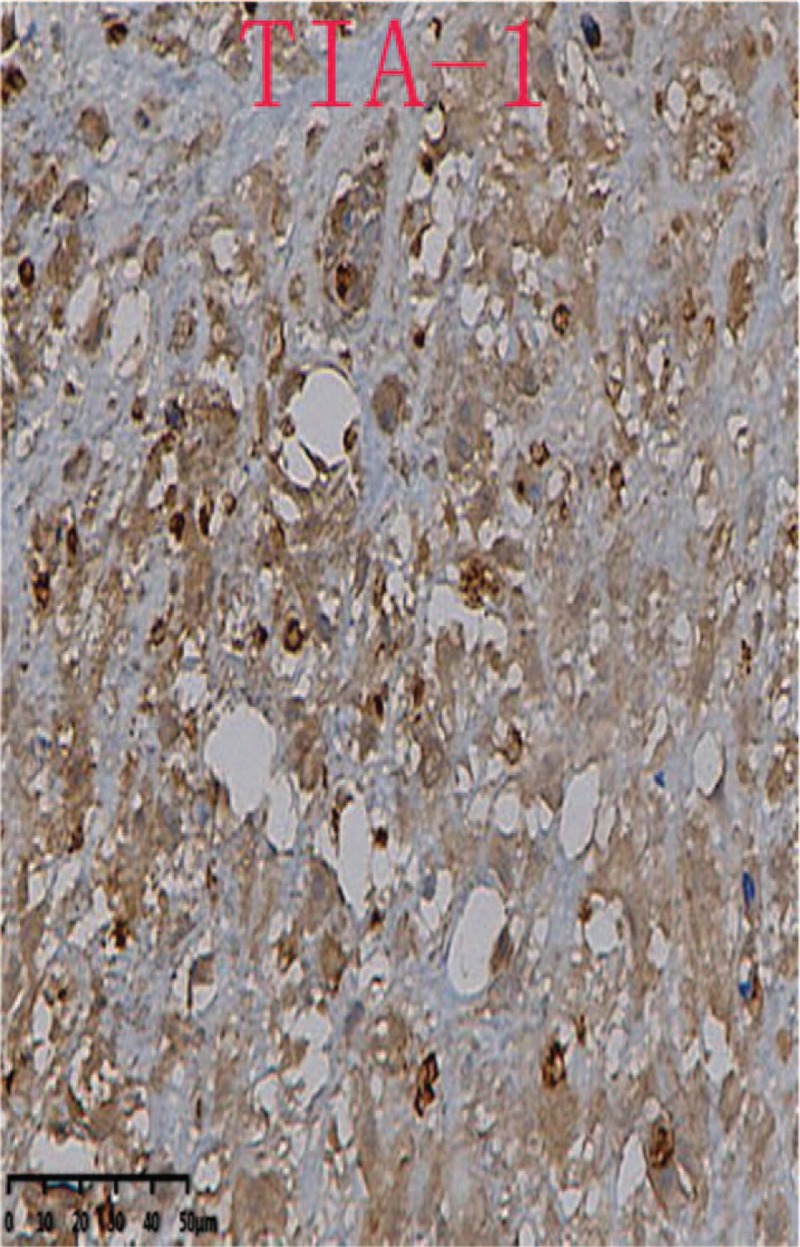
Granzyme B, TIA-1 (T-cell intracellular antigen) and Epstein–Barr virus–encoded mRNA (EBER) were positive. Ki-67 was 60%+, which indicated high cell proliferation (40×).

The myodynamia of both lower limbs were elevated to I degree with chest and back pain partly relieved after the operation. The patient had an intermittent fever which varied between 37.3°C and 38.9°C for a week, however, the result of blood and urine bacterial culture was negative. We suggested transferring the patient to oncology department to take the further treatment when the general vital signs were stable. The patient and his family members decided to discharge from hospital and take some Chinese medicine. During follow-up, the patient died about 3 months later.

## 3 Discussion

Diagnosis of such case might be challenging due to the lower prevalence. Moreover, the lesion location of our case was so atypical that it was difficult for us to draw an accurate conclusion at the early stage. As for this case, the clinical features just showed paravertebral and intraspinal occupying symptoms without any facial lesions. So if ENKTCL is suspected, histopathological and molecular biological examination of lesion is essential. Nasal type ENKTCL seems to be caused by EBV, and EBER in situ hybridization is the most reliable way to demonstrate the presence of EBV, which can be achieved from paraffin-embedded tissues.^[3]^ Meanwhile, this type of lymphoma arises as a consequence of malignant transformation of NK-cells (NKCs) which express markers CD56+, TIA-1, and lack T-cell receptor (*TCR*) gene rearrangements, the tumor cells typically have the immunophenotype of CD56+, CD2+, and cytoplasmic CD3+.^[4–6]^ Since CD56 is recognised as a NKC antigen, the tumor is termed NK T-cell lymphoma. However, the CD56 of our case was suspiciously positive, which was a bit different from common cases. That might be the reason of improper sampling location or staining problems. Expression of the CD3 antigen indicates that the tumor also comprises T lymphocytes as NKCs do not express this antigen (activated adult NKCs are negative for CD20, CD5, and surface CD3).^[4]^ The tumor cells will lack other T-cell antigens such as surface CD3, CD4, CD5, CD57, CD16, and CD20.^[7]^ Rarely, the tumor cells are positive for CD7, CD30, and CD45RO which is a T-cell marker.^[8]^ In our case, the expression of CD7 was positive. Nasal type ENKTCL do not have clonal rearrangements of the *TCR* genes thus allowing differentiation from other similar type disease such as enteropathy-associated T-cell lymphoma (which usually arises in the gastrointestinal tract).^[9]^ In addition, it has been reported that other cytotoxic molecules such as granzyme B and T-cell intracellular antigen-1 (TIA-1) (76.2% of all ENKTCL), can be expressed in this type of lymphoma.^[10]^ In conclusion, the final diagnosis should be made with the combination of clinical feature, PET-CT image, pathological morphology, immunophenotype and genetic features.

We took a posterior approach for excision of the sixth thoracic vertebral paravertebral and intraspinal occupying lesion because of the rapid increase of nerve compression symptoms, which got somewhat effects. Due to extranodal NK/T-cell lymphoma, nasal type is a rare and relatively newly recognised distinctive clinicopathologic entity in the WHO classification. Therefore, optimal treatment strategies and prognoses have not been fully defined yet. Most studies have shown that conventional chemotherapy followed by RT appears to be ineffective for the majority of patients. However, radiotherapy followed by, or combined with, chemotherapy may be the best initial treatment, perhaps with non-anthracycline-containing chemotherapy.^[11]^ In a retrospective study of 105 patients whose median ages were 52 years old (range 22–85), 76.2% of patients were Ann-Arbor stage I/II disease. The 5-year progression-free survival (PFS) and overall survival (OS) were 49.9% and 54.8%, respectively. Most stage I/II patients received combined chemoradiotherapy with anthracycline containing regimen, with overall response rate of 96.7%, complete response rate 86.9%, 5-year PFS 65%, and OS 72%. The relapse rate was 29.3% with a short median disease-free survival of 6.2 months. Among advanced stage patients, overall response rate was only 13.6%, with median PFS 2.3 months, and OS 4.8 months. Stages III/IV were unfavourable prognostic factors for PFS and OS by multivariate analyses,^[12]^ reflecting the significance of the early stage diagnosis from another hand. Theoretically, we think the combination of early stage diagnosis, chemotherapy and radiotherapy can improve the survival of patients through better local and systemic control of lymphoma.

Given the above, ENKTCL which originates from spine is especially rare, we did not give the accurate confirmation without immunophenotype analysis at the early stage. There are also limitations in the management of this case that we did not have a PET-CT image which could make a better diagnosis and our immunophenotype of CD56 is suspiciously positive. More cases like this may help us to find more significant clinical manifestations, immunohistopathological features and image manifestations laws of diagnosis. Orthopaedic surgeon should realize the possibility of this lesion location. The bad prognosis emphasizes the importance of early stage, accurate diagnosis and anti-tumor effects such as radiotherapy chemotherapy.

## Author contributions

**Conceptualization:** Jie Wang, Ning Li, Lei Xia.

**Formal analysis:** Jie Wang, Yonggang Fan, Ningning Yang.

**Investigation:** Jie Wang.

Lei Xia orcid: 0000-0003-1105-2239.
